# Supplementary material for: Achieving environmental stability in an atomically thin quantum spin Hall insulator via graphene intercalation
Source: Nat Commun. 2024 Feb 19;15:1486. doi: 10.1038/s41467-024-45816-9 (PMC10876696; doi:10.1038/s41467-024-45816-9)
Supplement: Supplementary file 1 — Supplementary Information [file 41467_2024_45816_MOESM1_ESM.pdf]

Supplementary Information - Achieving environmental stability in an atomically thin quantum spin Hall insulator via graphene intercalation

Cedric Schmitt,[1,2,*] Jonas Erhardt,[1,2,*] Philipp Eck,[2,3] Matthias Schmitt,[1,4] Kyungchan Lee,[1,2] Philipp Keßler,[1,2] Tim Wagner,[1,2] Merit Spring,[1,2] Bing Liu,[1,2] Stefan Enzner,[2,3] Martin Kamp,[1,5] Vedran Jovic,[6,7] Chris Jozwiak,[8] Aaron Bostwick,[8] Eli Rotenberg,[8] Timur Kim,[4] Cephise Cacho,[4] Tien-Lin Lee,[4] Giorgio Sangiovanni,[2,3] Simon Moser,[1,2] and Ralph Claessen[1,2,†]

[1] *Physikalisches Institut, Universität Würzburg, D-97074 Würzburg, Germany*
[2] *Würzburg-Dresden Cluster of Excellence ct.qmat, Universität Würzburg, D-97074 Würzburg, Germany*
[3] *Institut für Theoretische Physik und Astrophysik, Universität Würzburg, D-97074 Würzburg, Germany*
[4] *Diamond Light Source, Harwell Science and Innovation Campus, Didcot, OX11 0DE, United Kingdom*
[5] *Physikalisches Institut and Röntgen Center for Complex Material Systems, D-97074 Würzburg, Germany*
[6] *Earth Resources and Materials, Institute of Geological and Nuclear Science, Lower Hutt 5010, New Zealand*
[7] *MacDiarmid Institute for Advanced Materials and Nanotechnology, Wellington 6012, New Zealand*
[8] *Advanced Light Source, Lawrence Berkeley National Laboratory, Berkeley, CA 94720, USA*

(Dated: January 29, 2024)

CONTENTS

Supplementary Note 1: Graphene intercalation of bilayer indium 2

Supplementary Note 2: Cross sectional STEM of intercalated BL indium and indenene 4

Supplementary Note 3: Homogeneity of the intercalated indenene: STM characterization on the 500 nm scale 5

Supplementary Note 4: Electron density of graphene and substrate-induced carrier doping 6

Supplementary Note 5: Protection by graphene against immersion in water 8

Supplementary Note 6: Alkali metal doping 10

Supplementary Note 7: In-Si bond length by XSW photoemission 11

Supplementary Note 8: Topological classification by scanning tunneling spectroscopy 12

Supplementary References 15

* These authors have contributed equally
† e-mail: claessen@physik.uni-wuerzburg.de

SUPPLEMENTARY NOTE 1: GRAPHENE INTERCALATION OF BILAYER INDIUM

Following the well-established recipe of Ref. 1, the synthesis is initiated by sublimating the topmost Si atoms off the SiC(0001) substrate, leaving a C-rich buffer layer referred to as zero-layer graphene (ZLG, Supplementary Figure 1a$_1$). Low energy electron diffraction (LEED, Supplementary Figure 1a$_2$) reveals a characteristic $(6\sqrt{3}\times6\sqrt{3})$R30° fingerprint relative to the SiC(0001) (1×1) surface unit cell signalling a homogeneous ZLG coverage of the substrate. Corresponding angle-resolved photoemission (ARPES) measurements (Supplementary Figure 1a$_3$) show a broad and non-dispersive valence state at the K-point of the graphene Brillouin zone, affirming the covalent bonding to the underlying SiC that prevents ZLG from developing linear π-bands [1]. In a cyclic process of indium deposition and

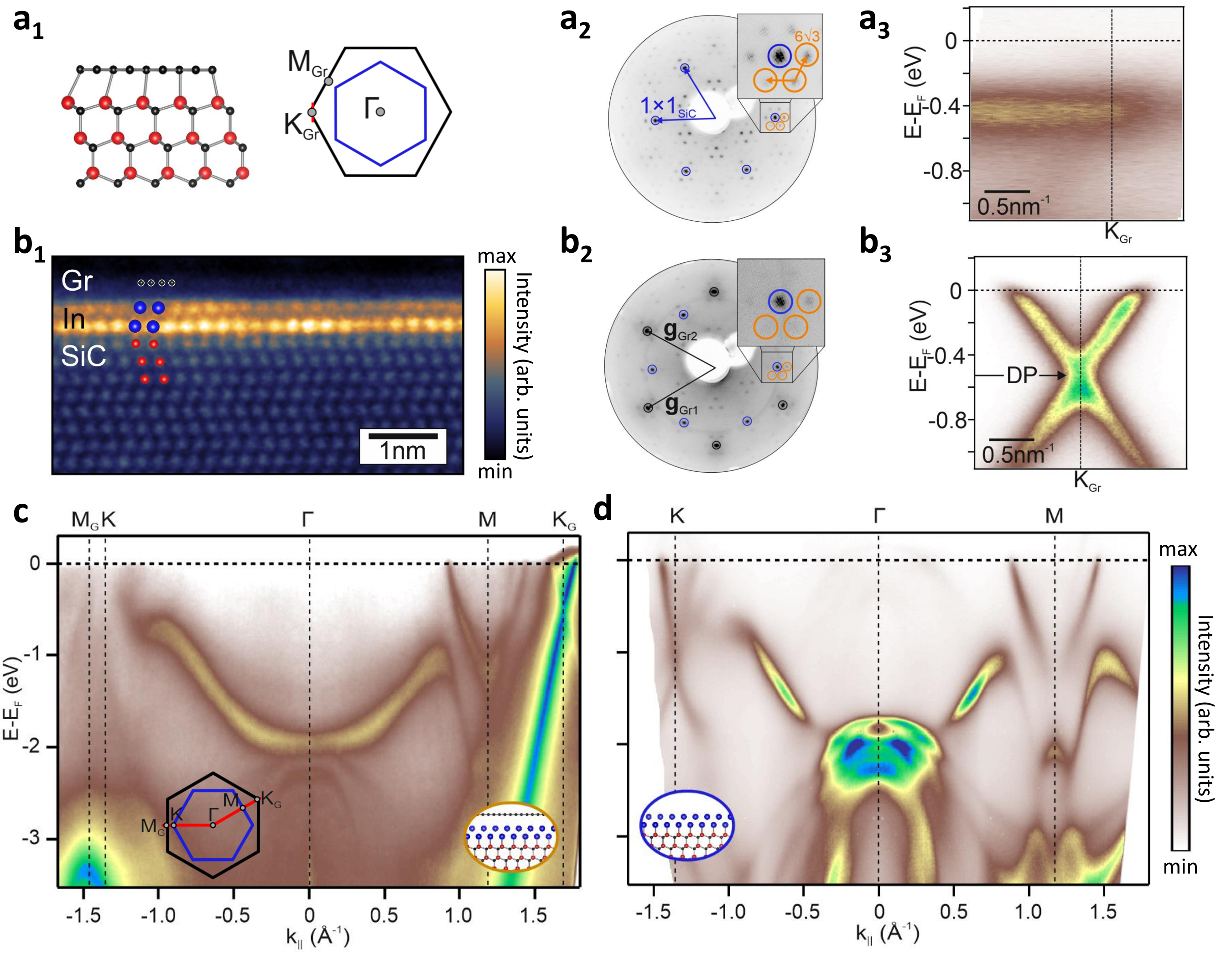

Supplementary Figure 1. **Graphene intercalation of 2 ML In and characterization by surface probes a$_1$** Schematic ball-and-stick model for buffer layer graphene and illustration of graphene (black) and indenene (blue) Brillouin zones. **b$_1$** Room-temperature (RT) STEM image of intercalated 2ML indium. **a$_2$,b$_2$** LEED images taken at 100 eV and (**a$_3$,b$_3$**) ARPES spectra around the graphene K-point K_{Gr} for **a** ZLG and **b** after indium bilayer intercalation taken along the path sketched in **a**. LEED images are normalized to SiC(0001) (1×1) spot (blue) intensities and show diffraction spots of $(6\sqrt{3}\times6\sqrt{3})$R30° periodicity (orange) [1] and graphene (black). **c,d** ARPES of **c** intercalated bilayer and **d** pristine bilayer indium on SiC(0001) taken at $h\nu$=46 eV (RT) and $h\nu$=21.2 eV (RT), respectively, along the path marked in the inset of **c**.

subsequent annealing, we replace the top layer carbon for indium as bonding partner to the substrate, hereby lifting the ZLG template from the subjacent SiC to form quasi-freestanding monolayer graphene (QFMG). The $(6\sqrt{3}\times6\sqrt{3})$R30° LEED signature of ZLG weakens significantly, while the diffraction spots of QFMG intensify and mark its decoupling from the substrate (Supplementary Figure 1b$_2$ and inset). ARPES now exhibits the characteristic π-band crossing of graphene (Supplementary Figure 1b$_3$), with the Dirac point (DP) at K (-0.5 eV) lying at slightly ($\sim$80 meV) higher

energies as compared to non-intercalated graphene on a ZLG buffer layer [1]. Representative scanning transmission electron micrographs (STEM) (Supplementary Figure $1b_1$) show two projected indium layers, each containing one In atom per Si site of the SiC surface. This puts forward a (1×1) adsorption geometry of 2 ML In on SiC(0001) and is corroborated by the absence of higher order diffraction spots in LEED (Supplementary Figure $1b_2$). At this point, our experimental results are reminiscent of recent experimental work on 2 ML In intercalated into a graphene SiC interface with three graphene top layers instead of one [2].

This is also true for the corresponding band structure accessed by ARPES (Supplementary Figure 1c), which we compare side by side to the pristine indium bilayer on SiC(0001) in Supplementary Figure 1d [3]. We recognize all ARPES features of the pristine bilayer phase to reappear in the intercalated counterpart, where we chose a different photon energy at which the indium 2D bands around are more prominent while SiC bands appear suppressed. In general, the bilayer band structure is very similar to indenene, yet shows additional metallic *sp* bands close to the M-point, which therefore serve as an ideal distinctive feature of the bilayer In coverage [3]. Overall, this demonstrates successful intercalation of 2 ML In whose transformation into the QSHI indenene via post-annealing is further elaborated in the main text.

SUPPLEMENTARY NOTE 2: CROSS SECTIONAL STEM OF INTERCALATED BL INDIUM AND INDENENE

In this section we demonstrate homogeneity of the intercalated 1 ML and 2 ML films on the lamella scale as well as proper alignment of the latter. All depicted STEM measurements were taken in high angle annular dark field (HAADF) mode. Prior to lamella preparation, we carefully characterize the sample by ARPES and LEED, thereby

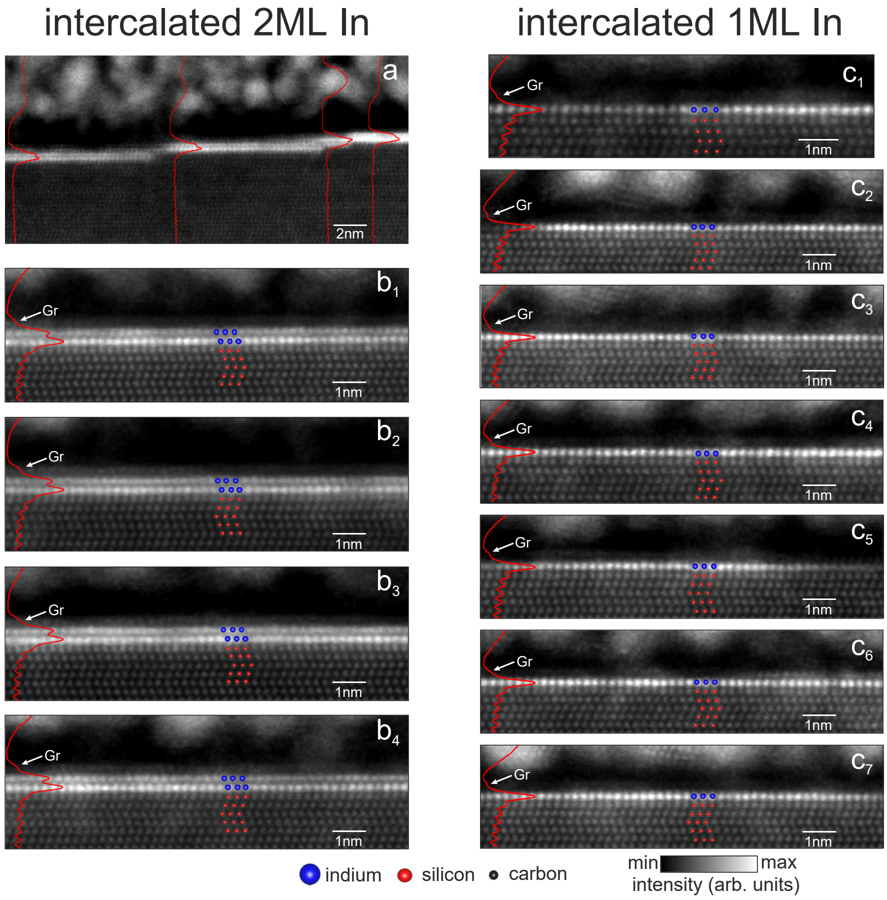

Supplementary Figure 2. **Cross sectional STEM measurements of intercalated bi- and monolayer indium. a** Step alignment within the lamella of 2 ML intercalated indium showing a consistent coverage of 2 ML indium on each terrace. Intensity profiles integrated over each terrace are depicted as insets (red). **b_1-b_4** STEM measurements of 2 ML intercalated indium at different positions of the lamella and overlaid structural model. The integrated intensity profile (red) emphasizes the weak graphene (Gr) signal. **c** STEM measurements of intercalated indenene, i.e., 1 ML indium reproduced for different terraces **c_1-c_7** within the lamella. The graphene cover is again most evident in the horizontally integrated intensity profile (red).

ensuring clear association of the band structure and STEM measurements. Nevertheless, misaligned surface steps in the lamella pose a risk of the In coverage being misinterpreted. We thus aligned the mostly parallel surface steps along the electron beam direction during preparation of the lamella. A representative STEM in Supplementary Figure 2a confirms this orientation and demonstrates an indium coverage of 2 ML on each terrace. This is reproduced in further STEM images taken at individual terraces (Supplementary Figure 2b) that consistently show the same positions of the indium rows as a related study on 2 ML intercalated indium [2]. We additionally depict horizontally integrated intensity profiles in each image in order to highlight the graphene signal (labeled Gr) whose weak signal is related to the Z-dependent sensitivity of this technique.

We proceed similarly for a sample hosting intercalated indenene. Representative STEM images in Supplementary Figure 2c show the In coverage consistently reduced to 1 ML In after the annealing treatment described in the main text, demonstrating a homogeneous film of intercalated indenene. Note that different SiC-terraces truncate the 4H-SiC(0001) in two different orientations evident in the sub-surface Si-rows in, e.g., Supplementary Figure 2b_1 and b_2.

SUPPLEMENTARY NOTE 3: HOMOGENEITY OF THE INTERCALATED INDENENE: STM CHARACTERIZATION ON THE 500 NM SCALE

Large scale homogeneity of the intercalated indenene film is further confirmed by representative scanning tunneling microscopy (STM) topography scans recorded in constant current (CC) mode and depicted in Supplementary Figure 2a,b and Fig. 2d of the main text. These images demonstrate a well-ordered and homogeneous film on 0.5μm sized terraces. Occasional small triangular pits are likely to form during the ZLG growth step and can be further reduced by optimizing the growth temperature. We readily identify these flat regions as intercalated indenene, either by reproducing the characteristic point spectroscopy depicted in Fig. 2g or by applying a lattice selective imaging method described in Fig. 2e,f of the main text, see zoom-ins in Supplementary Figure 2c,d. Note that the cloudy appearance of the intercalated indenene particularly evident in Supplementary Figure 2b,d is shaped by the inhomogeneous distribution of substrate doping and not an actual height modulation or bending of the lattice. This effect is well known for pristine indenene [3, 4] as well as other semiconducting surfaces [5]. The weak $\sqrt{3}$ modulation of the graphene lattice in Supplementary Figure 2c is related to quasi particle interference induced by a terrace outside the scanframe [6].

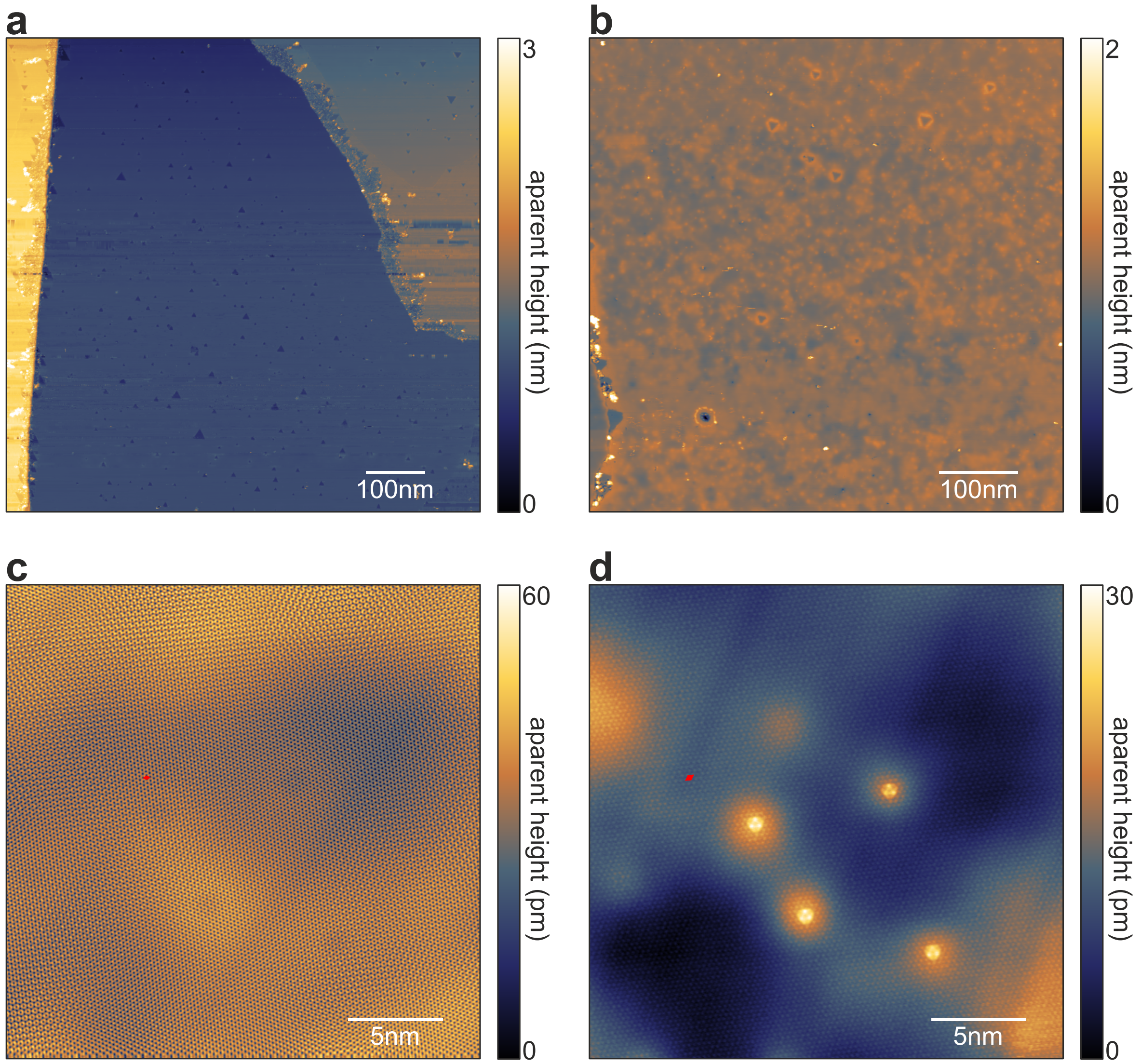

Supplementary Figure 3. **a,b** Large scale CC STM topography scans taken at (U_{set} =3.5 V, I_{set} =10 pA) taken after immersion in water and a mild degas. **c,d** Atomically resolved CC topography taken at the same position with **c** graphene sensitive parameters (U_{set} =95 mV, I_{set} =50 pA) as well as with **d** indenene sensitive tunneling parameters (U_{set} =790 V, I_{set} =150 pA). All STM measurements were recorded at 4.7 K.

SUPPLEMENTARY NOTE 4: ELECTRON DENSITY OF GRAPHENE AND SUBSTRATE-INDUCED CARRIER DOPING

Charge carrier concentrations of graphene and indenene (intercalated and pristine) are estimated from the Fermi surface area $n = k_F^2/\pi$. To rule out variations between substrates, we explicitly reused the exact same SiC substrate for the charge carrier analysis (and all data shown in Supplementary Figure 4). This was reproduced for physically different SiC substrates. We first investigate the band filling of graphene on intercalated bi- and monolayer indium. Note, the intercalated 2 ML and 1 ML bandmaps were taken at $h\nu$=46 eV, while the pristine counterparts were taken at $h\nu$=21.2 eV. The respective Fermi wavevectors k_F are extracted (i) by fitting Voigt profiles (error: half width at half maximum) to radially extracted momentum distribution curves (MDC) at E_F and centered at K, and (ii) by fitting Voigts profiles to the linear band dispersion within an energy interval around E_F (see red open circles and lines in Supplementary Figure 4a,b). Supplementary Figure 4a,b depicts ARPES measurements of the graphene K-point corresponding to 2 ML and 1 ML indium coverage, respectively. Quite remarkably, the transformation from 2 ML indium to 1 ML indenene redistributes charge within graphene and shifts the Dirac bands by 0.28 eV higher in energy compared to the 2 ML film. Similar band fillings upon post-annealing were already observed in Ref. 7, yet, were tentatively assigned to incomplete intercalation and a 2 ML phase, respectively. Related studies on epitaxial and quasi free-standing graphene on SiC(0001) identify charge transfer from the ZLG [8], the spontaneous polarization of the polar 4H-SiC [9, 10] and the space charge region formed in SiC [9] as the most relevant doping sources of graphene on SiC. These mechanisms also need to be carefully considered for intercalated indium and most likely contribute in different proportions for bi- and single layer indium, however, their scrutiny goes beyond the scope of this work and is left to future studies.

Turning to the band structure of the indenene, we observe a clear difference in the band occupation of the intercalated and pristine film. We quantify this observation by the energy shift ΔE that we extract by fitting Gaussian profiles to the EDCs of the pristine and intercalated indium band structure in the k-intervals ($-1\,\text{Å}^{-1}$ to $-0.5\,\text{Å}^{-1}$) and ($0.5\,\text{Å}^{-1}$ to $0.8\,\text{Å}^{-1}$). The EDC fit maxima in orange and red are overlaid with the ARPES bandmaps (see Supplementary Figure 4e-f) for the pristine and intercalated films, respectively. Note that, the observed ΔE is consistent with the shift of the Dirac bands at the K-point, depicted in Fig. 1a,b of the manuscript. The charge carrier concentrations as well as the results on ΔE are summarized in Supplementary Table 1. It is noteworthy that Δn_{In} of 2 ML In is highly sensitive to small changes in its large Fermi vector k_F, leading to a considerable error bar.

Supplementary Table 1. Intercalation induced graphene band filling n_G, indium band depletion Δn_{In} and indium band shift ΔE.

θ_{In}	n_G [10^{12} cm^{-2}]	Δn_{In} [10^{12} cm^{-2}]	ΔE [meV]
1 ML	3.44±0.31	-2.96±0.62	254±46
2 ML	16.0±1.1	/	52±50

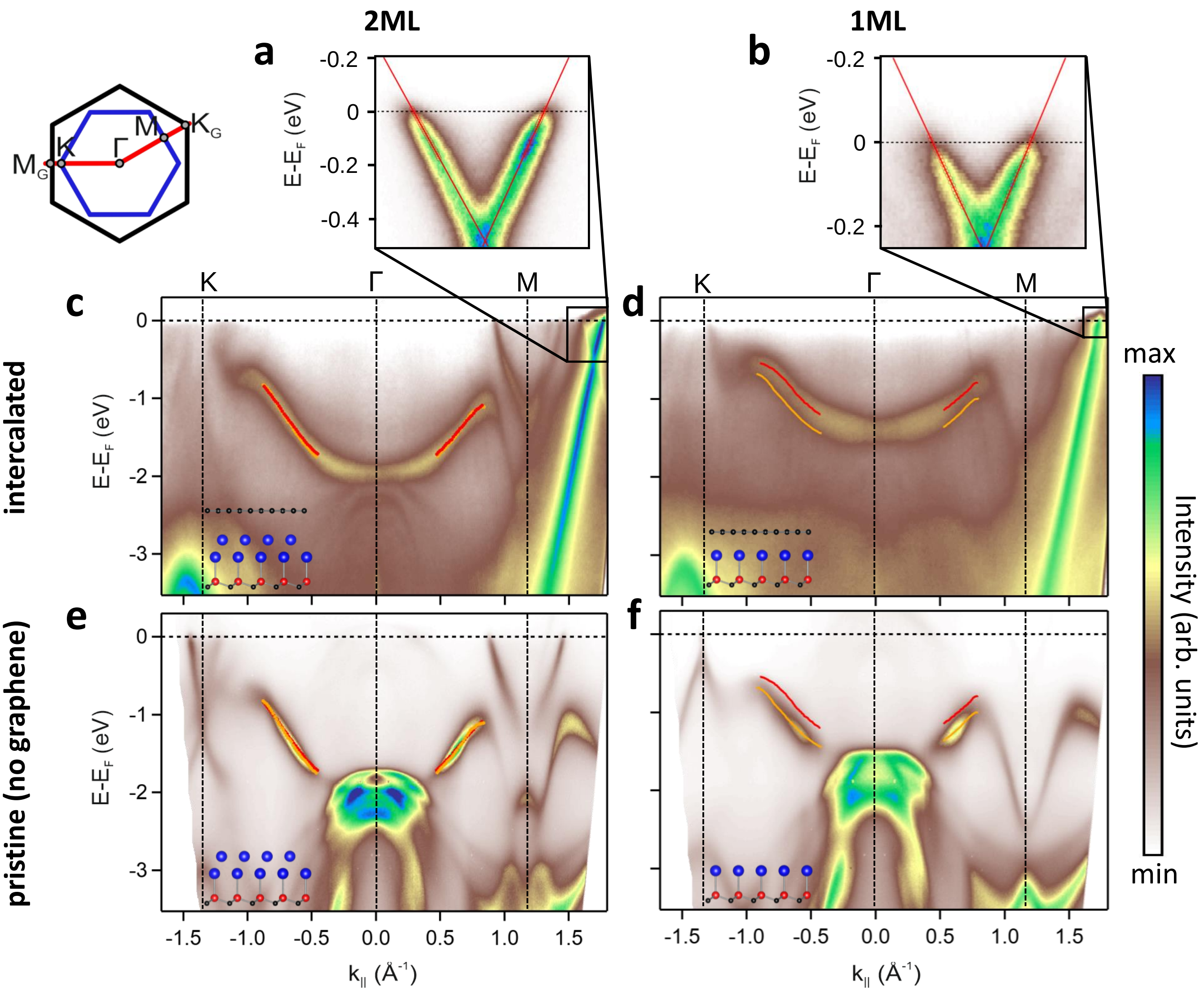

Supplementary Figure 4. **Charge carrier concentrations of intercalated and pristine bi- and monolayer In. a,b** ARPES spectra around the graphene K-point for **a** 2ML intercalated indium and **b** intercalated indenene as well as the linear fit of Voigt profiles within a small energy interval around E_F. **c-f** ARPES of **c** intercalated bilayer In, **d** intercalated monolayer In and **e** pristine bilayer In, **f** monolayer In on SiC(0001). The pristine data **e,f** were taken with $h\nu$=21.2 eV (RT), while the intercalated data **c,d** were taken at $h\nu$=46 eV (RT). The EDC fits in ΓM and ΓK direction are overlaid with the ARPES data representing pristine (orange) and intercalated (red) In films, respectively.

SUPPLEMENTARY NOTE 5: PROTECTION BY GRAPHENE AGAINST IMMERSION IN WATER

To evaluate if the intercalation process or immersion in water increases the degree of disorder compared to pristine indenene, we analyse the width of band features in energy dispersion curves (EDC) obtained by ARPES. We particularly, focus on EDC-spectra near the indenene K-point taken for pristine (a_3), as-grown intercalated indenene (a_2), as well as intercalated indenene after immersion in liquid water followed by a mild degas (a_1). Note, that the EDC of pristine indenene is plotted with an offset in energy to compensate the higher n-doping of the monolayer without graphene cover. EDCs (see Supplementary Figure 5b) are extracted at $k_{||} = 1.4\,\text{Å}^{-1}$ as indicated by a vertical line. We do not observe broadening of the Dirac bands in the investigated EDCs consequent to intercalation or an exposure to water. This shows that the same quality as in pristine indenene can be achieved by indenene intercalation, and that it is not reduced by the treatments discussed. To further underline the protective function of the graphene capping layer, we directly compare ARPES band maps before and after immersion in water, see Supplementary Figure 6. We find that all ARPES features are unaffected by this treatment, as is the band quality.

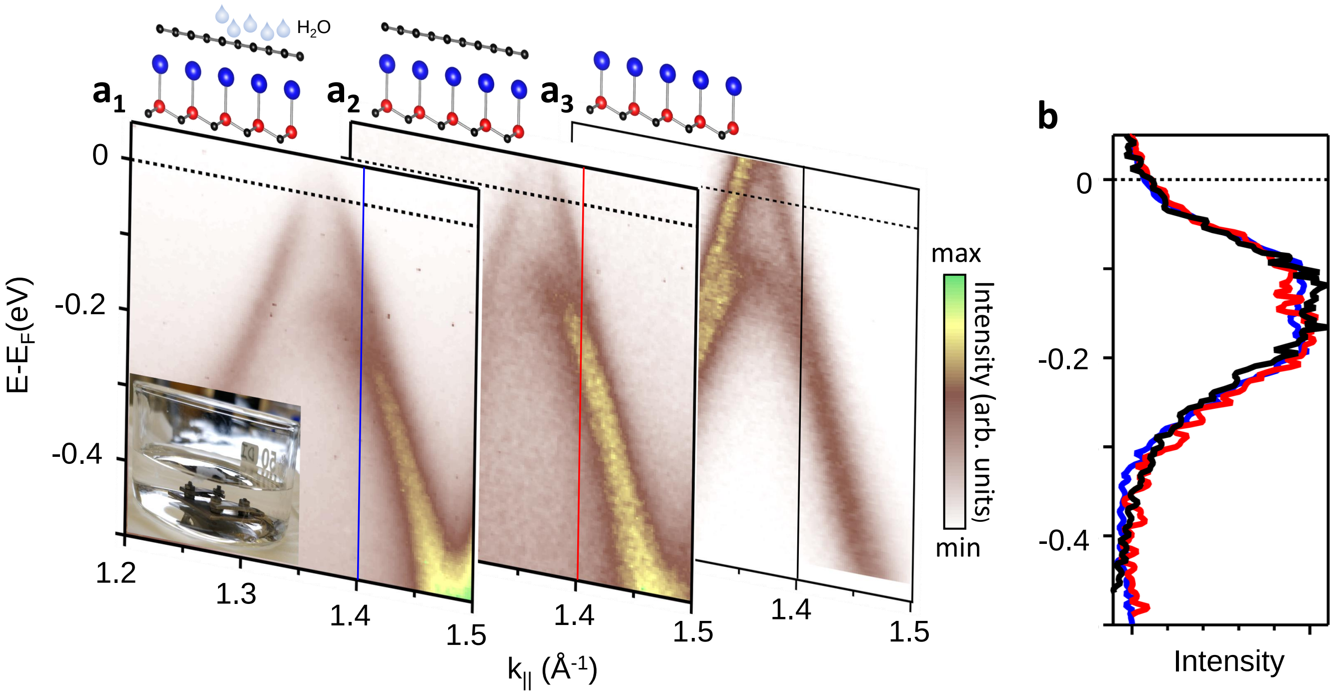

Supplementary Figure 5. **EDC analysis of (intercalated) indenene. a** ARPES K-point spectra of intercalated indenene $\mathbf{a_1}$ after immersion in water, $\mathbf{a_2}$ after growth and $\mathbf{a_3}$ of pristine indenene that is offset in energy to compensate differences in the band filling. **b** EDCs extracted at $k_{||} = 1.4\,\text{Å}^{-1}$ from **a** after water exposure (blue) and after growth of intercalated indenene (red) and pristine indenene (black). All data were recorded at RT. The photo in the inset of $\mathbf{a_1}$ shows the investigated sample during exposure to water.

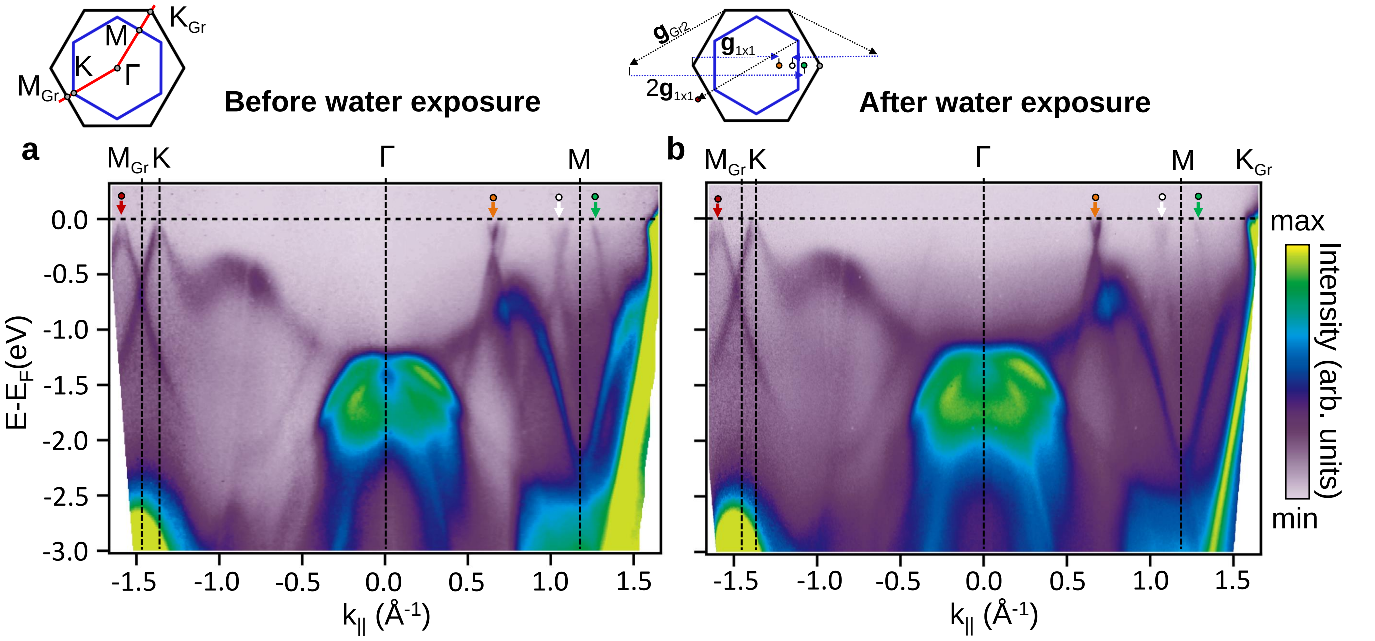

Supplementary Figure 6. **The band structure of intercalated indenene before and after exposure to water.** ARPES measurements of intercalated indenene **a** before and **b** after immersion in water and a mild degas. The data were taken at RT with $h\nu$=21.2 eV. The top row illustrations show the Brillouin zones of indenene (blue) and graphene (black) and the high symmetry k-path (red) along which the ARPES data are shown. Graphene and indenene band replicas in **b** that are consistent with electron diffraction off the In/SiC (orange) or graphene lattice (red) and replicas consistent with multiple scattering (white, green) are shown in the sketch top right.

SUPPLEMENTARY NOTE 6: ALKALI METAL DOPING

Albeit the substrate choice brings intercalated indenene close to charge neutrality, existing samples remain p-type, denying direct access to the band gap in ARPES. A possible solution is the surface deposition of alkali metals, which is an established technique to increase the band filling, however, sometimes comes with the downside to change the band structure itself due to the electric dipole field normal to the surface that is created by the positively charged alkali ions [11]. For the Dirac states of intercalated indenene, the latter effect can be ruled out by symmetry considerations, which show that an out-of-plane electric field has no influence on these in-plane $p_{+/-}$ states and thus neither alters band gap nor the topology of indenene [12]. The envisaged electron doping is indeed observed in the doping series of intercalated indenene presented in Fig. 4a of the main text, where repeated potassium deposition reveals the conduction band minimum and thus the band gap.

In this section, we complement these data by the graphene K-point as well as the In 4*d* core level upon potassium deposition. The ARPES measurements in Supplementary Figure 7a were taken before and after the K-doping study depicted in Fig. 4a,b of the main text and show both indenene and graphene K-point simultaneously, recorded approximately along the red path sketched in Supplementary Figure 7. For indenene, we find a consistent energy shift (-0.29 eV) to higher binding energies of both valence bands as well as In 4*d* core level (Supplementary Figure 7b). Next to the latter, at approximately -19 eV, a K 3*p*-related shoulder appears after doping confirming the presence of potassium. Even if K-doping is significantly more effective for graphene, evident in an energy shift of the Dirac point of approximately -0.5 eV, we do not observe additional changes to its Dirac bands at the given resolution.

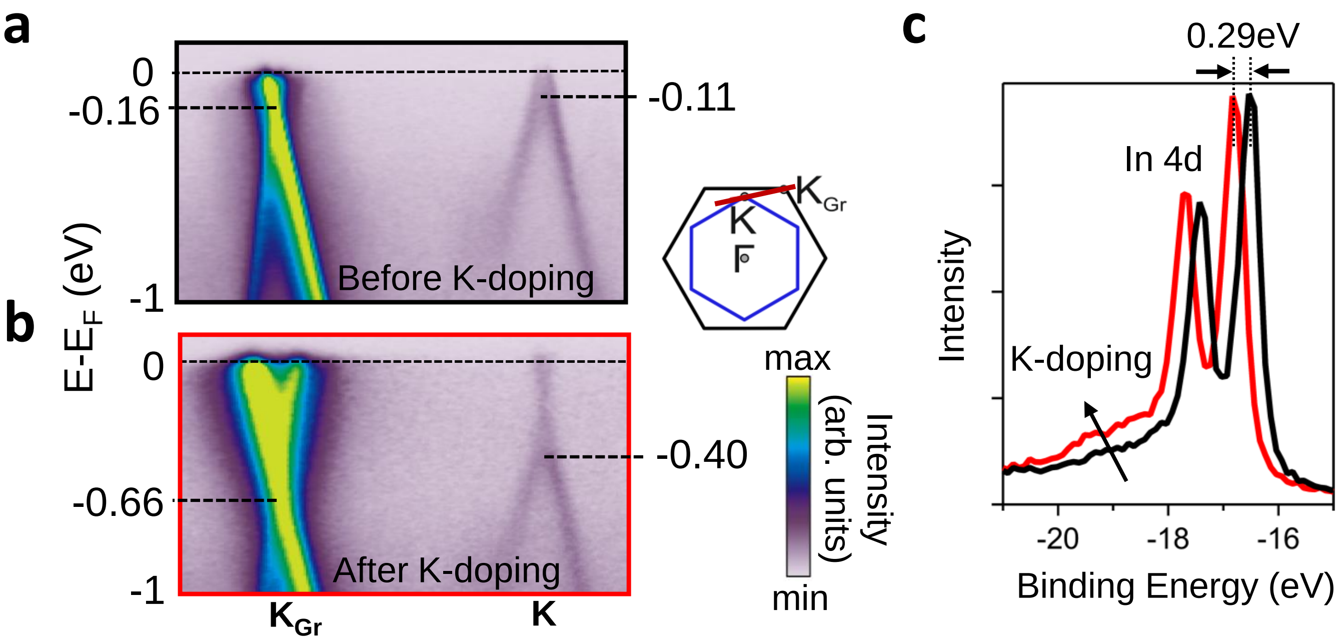

Supplementary Figure 7. **Potassium doping of intercalated indenene. a,b** ARPES spectra of the graphene and indenene K-points **a** before and **b** after potassium deposition presented in Fig. 4a,b of the main text. Data are taken with hν=90 eV and approximately along the path sketechd between the Brillouin zones of indenene (blue hexagon) and graphene (black hexagon). **c** Ultraviolet photoelectron spectra of In 4*d* and K 3*p* core-levels before (black) and after (red) K-deposition excited by hν=90 eV photons.

SUPPLEMENTARY NOTE 7: IN-SI BOND LENGTH BY XSW PHOTOEMISSION

Normal incidence X-ray standing wave (XSW) measurements were performed in analogy to our preceding experiments [4] at beamline I09 at Diamond Light Source, England, in UHV environment at RT. The samples were prepared and characterized by ARPES in our home lab before shipping them in N_2 atmosphere (intercalated indenene) or *in vacuo* (pristine indenene) in a UHV suitcase with base pressure below 10^{-9} mbar. The XSW field is created by Bragg reflection from the SiC(0004) plane. Tuning the photon energy across the Bragg condition shifts the spatial position of the standing wavefield with respect to the substrate lattice planes, thereby producing a characteristic photon energy dependent photoelectron yield that depends on the relative distance - the so-called coherent position PH - of the atomic species under investigation to the SiC(0004) plane (spacing of 2.52 Å [13]). We apply a well-established analysis procedure [14] to obtain PH of the silicon, graphene and indenene layers from Si 2s, C 1s (graphene), In 3d (intercalated) and In 4d (pristine) core-level photoelectron yields. Results presented in Supplementary Table 2 are consistent with low temperature (20 − 30 K) In 3d and In 4d values of pristine indenene presented in Ref. [4].

Supplementary Table 2. Coherent positions PH (in multiples of the SiC(0004) plane spacing [13]) of intercalated and pristine indenene determined by room-temperature XSW [14] for silicon, indium and graphene-related carbon. The error of PH values is estimated to be 0.01. For physical distances we add multiples of the SiC(0004) plane spacing (see brackets) that are motivated by STEM images.

indenene	PH_{In}	PH_{Si}	$PH_{G\text{-}C}$
pristine	0.06 (+1)	0	/
intercalated	0.09 (+1)	0	0.42 (+2)

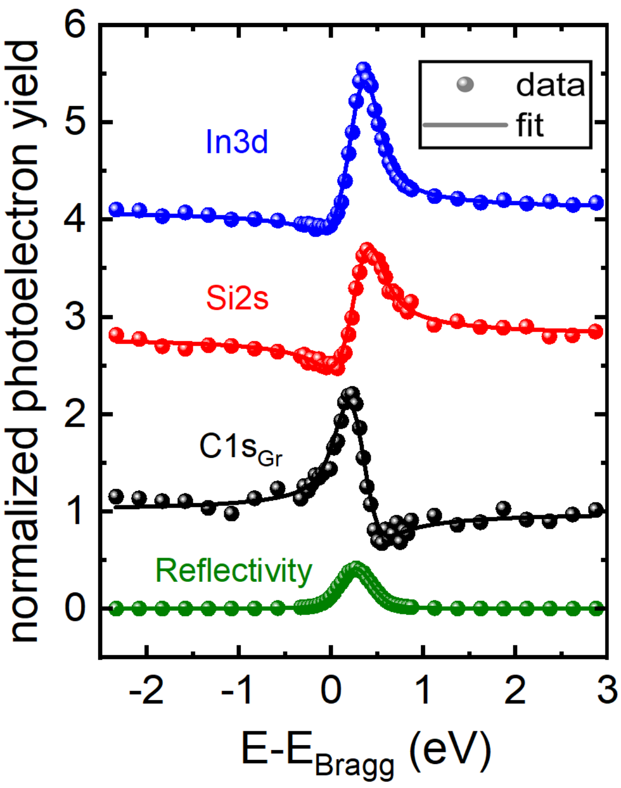

Supplementary Figure 8. **Normal incidence X-ray standing wave.** XSW yield curves of the In 3d, Si 2s, C 1s_{Gr} and the X-ray reflectivity lead to the coherent positions PH listed Supplementary Table 2.

SUPPLEMENTARY NOTE 8: TOPOLOGICAL CLASSIFICATION BY SCANNING TUNNELING SPECTROSCOPY

Indenene has a well established connection between its non-trivial topology and an alternating L_z-sequence of its Dirac states [4, 12]. A potential trivial phase on the other hand, would induce a non-alternating L_z-sequence for which both valence (conduction) bands at K(K') share the same L_z eigenvalue, respectively. Via wavefunction interference the L_z-sequence translates into an alternating (non-trivial) or non-alternating (trivial) charge localization in the unit cell, directly accessible to dI/dV mapping in STS [4]. To probe this topological fingerprint in intercalated indenene, we perform constant height (CH) dI/dV mapping of the second (VB-1) and first indenene valence band (VB), whose energy position is readily identified in a preceding characterization in ARPES depicted in Supplementary Figure 9a. Other than the conduction bands, these are only marginally overshadowed by the linearly increasing graphene local density of states ($LDOS_{Gr} \propto |U_{Bias} - U_{DP_{GR}}|$) as they lie within a small energy window (orange in Supplementary Figure 9a) around graphene Dirac point DP_{Gr}, where $LDOS_{Gr}$ is small, see Supplementary Figure 9a and, e.g., Fig. 3b of the main text.

With the help of Supplementary Figure 9a,b, we select U_{Bias} =-100 mV for probing VB-1 and indeed, find a clear modulation of the graphene lattice (Supplementary Figure 9c_1) that indicates contributions from additional periodicities. These are pinpointed by the fast Fourier transform (FFT) depicted in Supplementary Figure 9c_2 and correspond to graphene (encircled black), indenene (encircled blue) and their $(6\sqrt{3} \times 6\sqrt{3})R30^\circ$ moiré unit cell. An inverse FFT of the indenene FFT-components thus isolates its dI/dV contribution to the total signal, thereby revealing the LDOS of the buried indenene. Supplementary Figure 9c_3 depicts the correspondingly FFT-filtered indenene lattice at -100 mV corresponding to VB-1, where the charge maximum occurs at position A of the unit cell, see Ref. 4. To solidify this position for VB-1, we select -50 mV as another VB-1 sensitive bias voltage (see Supplementary Figure 9a) and still find the charge maximum at position A, see Supplementary Figure 9d. In contrast, raising U_{Bias} above VB-1, such as at 50 mV (Supplementary Figure 9a), leaves only contributions of VB to the tunneling signal and causes the charge maximum to switch to position B of the unit cell (Supplementary Figure 9e_3). This A-B switch in the valence bands establishes the non-trivial topology of intercalated indenene. We confirm the charge maximum of VB to reside at B by probing at 100 mV, see Supplementary Figure 9f.

To demonstrate that this A-B-relocation of the charge maximum is indeed related to the valence bands, we repeat this experiment for less p-type indenene in the right column of Supplementary Figure 9, where both ARPES spectra (panel g) and STS spectra (panel h) are shifted by ≈ 150 meV. Tracing the charge maximum for related U_{Bias} in Supplementary Figure 9i-l, we find the A-B switch well reproduced and clearly connected to the energy position of VB-1 and VB, thus unambiguously demonstrating the non-trivial topology of intercalated indenene.

Note that we reproduced this finding more than 10 times on four different samples. Supplementary Fig. 10 shows extended data on Fig. 4e,f of the main text, containing also the unfiltered dI/dV measurement as well as their FFT magnitude spectrum. In the analysis we carefully correct the STM scan frame drift by using the graphene honeycombs of successive dI/dV scans. For the FFT-filtering of the dI/dV maps we apply the inverse FFT to all frequencies (and phases) within a radius of 0.12 $Å^{-1}$ of the main indenene (1×1) frequencies.

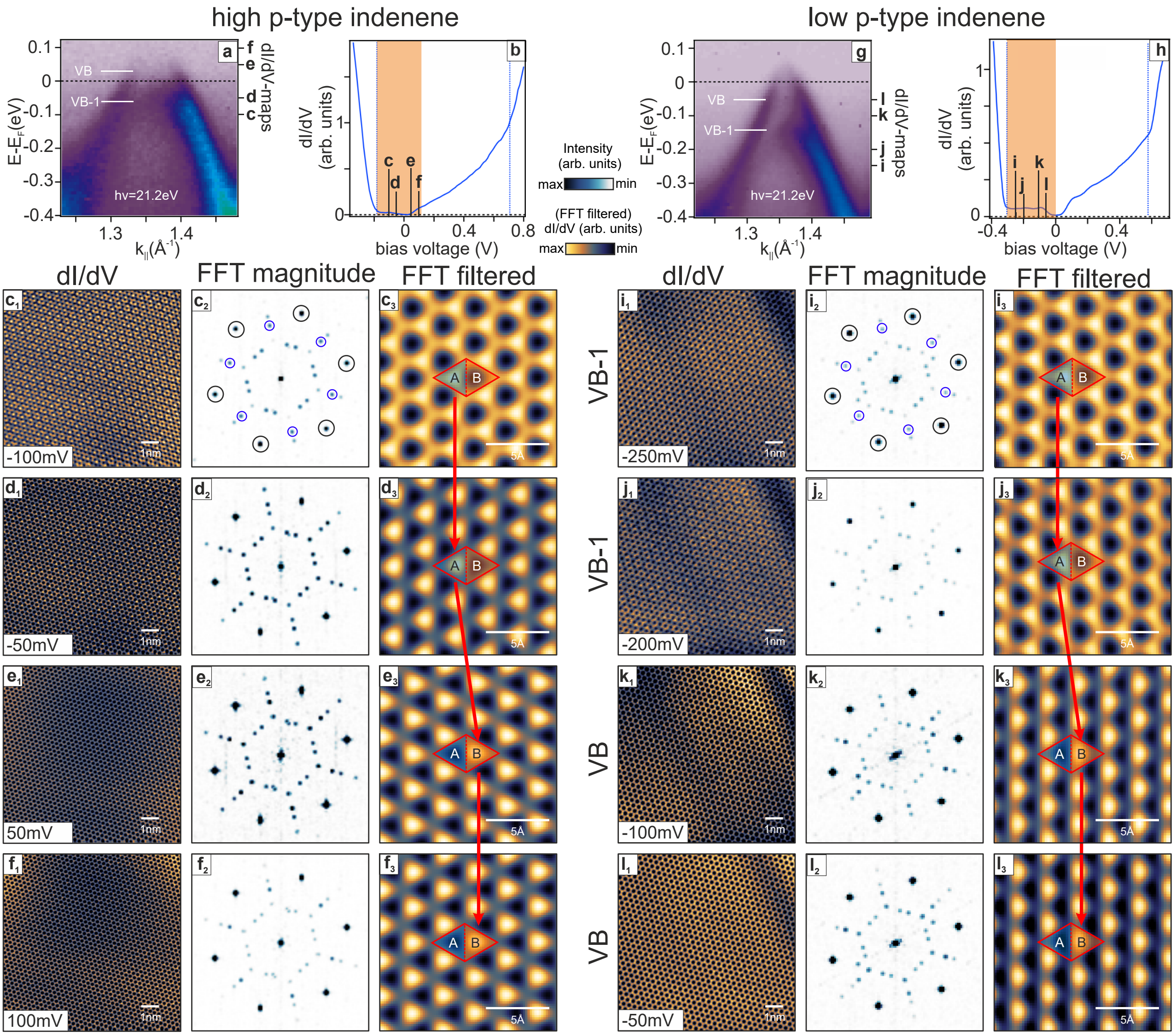

Supplementary Figure 9. **Experimental fingerprint of non-trivial topology of intercalated indenene: Charge localization accessed by STS. a** ARPES measurement (RT) and **b** STS point spectroscopy (U_{set}=850 mV, I_{set}=50 pA) of high p-type intercalated indenene and approximate energy positions of the dI/dV maps shown in **c-f**. The orange region in STS marks low graphene LDOS. High-doped **c-f** and low-doped **i-l** p-type intercalated indenene dI/dV measurements; Column 1: dI/dV maps taken in CH mode using a standard lock-in technique with modulation frequency of 971 Hz and modulation voltage V_{rms} =12 mV (time constant 1 ms). Column 2: FFT magnitude spectrum of the corresponding dI/dV map with indenene (encircled blue) and graphene frequencies (encircled black). Column 3: Inverse FFT (zoom-in) of indenene frequencies and phases. Arrows highlight the position of the charge maximum in the unit cell revealing a switch from A to B when changing from VB-1 to VB. **g,h** ARPES measurement (RT) and STS point spectroscopy (U_{set}=500 mV, I_{set}=0.5 nA) of low-doped p-type intercalated indenene and indicated energy positions of the dI/dV maps shown in **i-l**. All STM/STS data were recorded at 4.7 K.

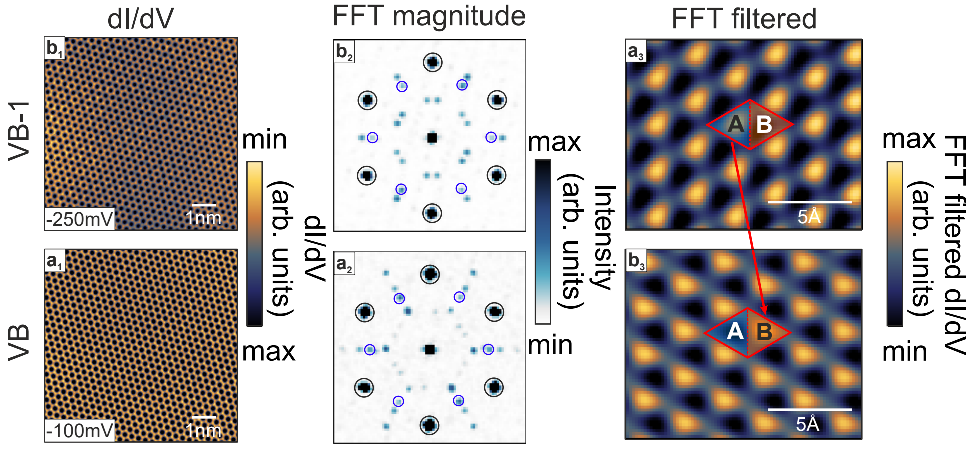

Supplementary Figure 10. **Extended data on filtered dI/dV maps shown in Fig. 4e,f: Charge localization accessed by STS. a,b** dI/dV measurements of intercalated indenene taken in CH mode and at **a** -250mV and **b** -100mV. Column 1: dI/dV maps taken in CH mode using a standard lock-in technique with modulation frequency of 971 Hz and modulation voltage $V_{\rm rms}$ =15 mV (time constant 1 ms). Column 2: FFT magnitude spectrum of the corresponding dI/dV map with indenene (encircled blue) and graphene frequencies (encircled black). Column 3: Inverse FFT (zoom-in) of indenene frequencies and phases. The red arrow highlights the position of the charge maximum in the unit cell revealing a switch from A to B when changing from VB-1 to VB. All STM/STS data were recorded at 4.7 K.

SUPPLEMENTARY REFERENCES

[1] C. Riedl, C. Coletti, and U. Starke, Structural and electronic properties of epitaxial graphene on SiC(0001): A review of growth, characterization, transfer doping and hydrogen intercalation, J.Phys. D **43**, 374009 (2010).
[2] N. Briggs, B. Bersch, Y. Wang, J. Jiang, R. J. Koch, N. Nayir, K. Wang, M. Kolmer, W. Ko, A. De La Fuente Duran, S. Subramanian, C. Dong, J. Shallenberger, M. Fu, Q. Zou, Y.-W. Chuang, Z. Gai, A.-P. Li, A. Bostwick, C. Jozwiak, C.-Z. Chang, E. Rotenberg, J. Zhu, A. C. T. van Duin, V. Crespi, and J. A. Robinson, Atomically thin half-van der Waals metals enabled by confinement heteroepitaxy, Nat. Mater. **19**, 637 (2020).
[3] J. Erhardt, M. Bauernfeind, P. Eck, M. Kamp, J. Gabel, T.-L. Lee, G. Sangiovanni, S. Moser, and R. Claessen, Indium epitaxy on sic(0001): A roadmap to large scale growth of the quantum spin Hall insulator indenene, J. Phys. Chem. C **126**, 16289 (2022).
[4] M. Bauernfeind, J. Erhardt, P. Eck, P. Thakur, J. Gabel, T.-L. Lee, J. Schäfer, S. Moser, D. D. Sante, R. Claessen, and G. Sangiovanni, Design and realization of topological Dirac fermions on a triangular lattice, Nat. Commun. **12**, 5396 (2021).
[5] P. H. Weidlich, R. E. Dunin-Borkowski, and P. Ebert, Quantitative determination of local potential values in inhomogeneously doped semiconductors by scanning tunneling microscopy, Phys. Rev. B **84**, 085210 (2011).
[6] G. M. Rutter, J. N. Crain, N. P. Guisinger, T. Li, P. N. First, and J. A. Stroscio, Scattering and interference in epitaxial graphene, Science **317**, 219 (2007).
[7] H. Kim, N. Tsogtbaatar, B. Tuvdendorj, A. Lkhagvasuren, and J. Seo, Effects of two kinds of intercalated In films on quasi-free-standing monolayer graphene formed above SiC(0001), Carbon **159**, 229 (2020).
[8] S. Kopylov, A. Tzalenchuk, S. Kubatkin, and V. I. Fal'ko, Charge transfer between epitaxial graphene and silicon carbide, Appl. Phys. Lett. **97**, 112109 (2010).
[9] S. Mammadov, J. Ristein, R. J. Koch, M. Ostler, C. Raidel, M. Wanke, R. Vasiliauskas, R. Yakimova, and T. Seyller, Polarization doping of graphene on silicon carbide, 2D Mater. **1**, 035003 (2014).
[10] J. Ristein, S. Mammadov, and T. Seyller, Origin of doping in quasi-free-standing graphene on silicon carbide, Phys. Rev. Lett. **108**, 246104 (2012).
[11] T. Ohta, A. Bostwick, T. Seyller, K. Horn, and E. Rotenberg, Controlling the electronic structure of bilayer graphene, Science **313**, 951 (2006).
[12] P. Eck, C. Ortix, A. Consiglio, J. Erhardt, M. Bauernfeind, S. Moser, R. Claessen, D. Di Sante, and G. Sangiovanni, Real-space obstruction in quantum spin Hall insulators, Phys. Rev. B **106**, 195143 (2022).
[13] M. Stockmeier, R. Müller, S. A. Sakwe, P. J. Wellmann, and A. Magerl, On the lattice parameters of silicon carbide, J. Appl. Phys. **105**, 033511 (2009).
[14] D. P. Woodruff, Surface structure determination using X-ray standing waves, Rep.Prog. Phys. **68**, 743 (2005).
